# Anti-Apoptotic and Antioxidant Activities of the Mitochondrial Estrogen Receptor Beta in N2A Neuroblastoma Cells

**DOI:** 10.3390/ijms22147620

**Published:** 2021-07-16

**Authors:** Ioannis Tsialtas, Achilleas Georgantopoulos, Maria E. Karipidou, Foteini D. Kalousi, Aikaterini G. Karra, Demetrios D. Leonidas, Anna-Maria G. Psarra

**Affiliations:** Department of Biochemistry and Biotechnology, University of Thessaly, 81500 Larissa, Greece; tsialtasj@gmail.com (I.T.); ageorgant@bio.uth.gr (A.G.); mkaripidou@uth.gr (M.E.K.); fokalous@bio.uth.gr (F.D.K.); aikaterini.g.karra@gmail.com (A.G.K.); ddleonidas@bio.uth.gr (D.D.L.)

**Keywords:** estrogen receptor beta, mitochondria, apoptosis, oxidative stress, neuroprotection

## Abstract

Estrogens are steroid hormones that play a crucial role in the regulation of the reproductive and non-reproductive system physiology. Among non-reproductive systems, the nervous system is mainly affected by estrogens due to their antioxidant, anti-apoptotic, and anti-inflammatory activities, which are mediated by membranous and nuclear estrogen receptors, and also by non-estrogen receptor-associated estrogen actions. Neuronal viability and functionality are also associated with the maintenance of mitochondrial functions. Recently, the localization of estrogen receptors, especially estrogen receptor beta, in the mitochondria of many types of neuronal cells is documented, indicating the direct involvement of the mitochondrial estrogen receptor beta (mtERβ) in the maintenance of neuronal physiology. In this study, cell lines of N2A cells stably overexpressing a mitochondrial-targeted estrogen receptor beta were generated and further analyzed to study the direct involvement of mtERβ in estrogen neuroprotective antioxidant and anti-apoptotic actions. Results from this study revealed that the presence of estrogen receptor beta in mitochondria render N2A cells more resistant to staurosporine- and H_2_O_2_-induced apoptotic stimuli, as indicated by the reduced activation of caspase-9 and -3, the increased cell viability, the increased ATP production, and the increased resistance to mitochondrial impairment in the presence or absence of 17-β estradiol (E2). Thus, the direct involvement of mtERβ in antioxidant and anti-apoptotic activities is documented, rendering mtERβ a promising therapeutic target for mitochondrial dysfunction-associated degenerative diseases.

## 1. Introduction

Estrogens play a central role in the regulation of the biochemical processes related to female reproduction [1] and non-reproductive tissue physiology [2], but they also act on male reproductive tissue [3]. Thus, a wide variety of biological actions, including differentiation, cell proliferation, apoptosis, antioxidant defense, and inflammation, are regulated by the main estrogenic hormone 17-β estradiol (E2) [4]. The classical mechanism of estrogen actions is mainly mediated by the nuclear estrogen receptors (ERs), estrogen receptor alpha (ERα) and estrogen receptor beta (ERβ), which function as ligand-dependent transcription factors, regulating the transcription of target genes containing the consensus estrogen response element (ERE) in their promoter regions. Estrogen binding to ERs triggers their nuclear translocation, homo- or heterodimerization, and binding to specific EREs in the nuclear DNA. ERs can also be found in the nucleus independently of the presence of the hormone [5,6]. Estrogen-activated nuclear ERs can also regulate non-ERE dependent transcription via interactions with other transcription factors, such as AP-1, affecting positively or negatively the expression of their target genes. In addition to the classical nuclear actions of estrogen, rapid membrane-initiated actions could also take place, which are mediated via plasma membrane-associated mERα, mERβ and G-protein coupled ERs (GPER, GPER1) [7,8,9]. Estrogen actions that proceed independently of estrogen receptors have also been reported [9]. Moreover, mitochondrial localization of ERs has been observed in a variety of cell types, including neuronal cells [10,11,12]. The biochemical mechanisms of the direct actions of estrogen in mitochondria, via their cognate receptors, is under investigation and many aspects of these actions remain to be verified and further explored. Mitochondrial estrogen receptors, especially estrogen receptor beta, is proposed to act as a direct target of estrogens, contributing to the estrogen-dependent orchestration and coordination of nuclear and mitochondrial functions, affecting mitochondrial transcription, reactive oxygen species (ROS) production, and apoptosis [6,13,14].

Protective actions of estrogens for human physiology are well documented [15,16,17,18]. Especially for the nervous system, a significant amount of evidence revealed estrogen’s neuroprotective actions. Thus, estrogen is proposed to promote neuronal survival, to control neuronal excitability and synaptic plasticity, and to regulate neuronal differentiation, brain development, neuronal homeostasis, and astroglial and microglial inflammatory responses [19,20]. Both nuclear and membrane estrogen receptors participate in estradiol neuroprotective actions, among others, via the control of aromatase, b-cell lymphoma 2 (BCL-2) family member and cytokine synthesis, glycogen synthase kinase-3β (GSK3β) inhibition, glutamate uptake, and calcium and ROS level regulation [19,20,21]. ERs actions also involve interactions with alternative pathways orchestrated by other neuroprotective factors, namely insulin growth factor-1, brain derived neurotrophic growth factor, and regulatory factors of WNT (Wingless-Int) and Notch signaling [19]. Thus, estradiol is proposed as a neuroprotective factor in many neurodegenerative diseases such as Alzheimer’s disease, also affecting amyloid beta synthesis and toxicity [22].

It is also widely accepted that neurons critically depend on mitochondrial function to ensure membrane excitability and to execute the complex processes of neurotransmission and plasticity [23]. Thus, mitochondrial dysfunction which is accompanied with increased ROS production and oxidative damage is associated with numerous neurodegenerative disorders [24].

To explore whether mitochondrial estrogen receptor beta is directly involved in neuroprotection against apoptotic and oxidative stress stimuli, neuroblastoma N2A cells stably overexpressing a mitochondrial targeted ERβ fused with the green fluorescence protein were generated (N2AmtGFPERβ). N2A cells stably overexpressing a mitochondrial-targeted GFP were also produced and used as control cells. Comparative studies to investigate the mtERβ effect on staurosporine- and H_2_O_2_-induced apoptosis and oxidative stress, in the presence or absence of E2, were performed, applying MTT assay, mitochondrial staining assessment via confocal microscopy, Western blot analysis of mitochondrial dependent apoptosis caspases, and ATP measurements. Our results showed that mtΕRβ expression renders N2A neuroblastoma cells more resistant against apoptotic and oxidative stress stimuli. Thus, the direct antioxidant and anti-apoptotic actions of mtERβ are documented, rendering mitochondrial ERβ a promising therapeutic target for mitochondrial dysfunction-associated degenerative diseases.

## 2. Results

### 2.1. Generation of N2A Cells Stably Overexpressing a Mitochondrial-Targeted GFPERβ Protein

Focusing on the elucidation of the role of the mitochondrial ERβ in mitochondrial functions, and particularly in the regulation of the mitochondrial-dependent apoptosis and antioxidant stress, N2A cells stably overexpressing a mitochondrial-targeted human ERβ fused with the green fluorescence protein (GFP) at its *n* terminus was generated (N2AmtGFPERβ), as described in the experimental section. N2A cells stably overexpressing a mitochondrial-targeted GFP (N2AmtGFP) were also produced and used as a control. Characterization of single colonies of the N2AmtGFPERβ and N2AmtGFP cells was performed as follows.

**Figure 1:**
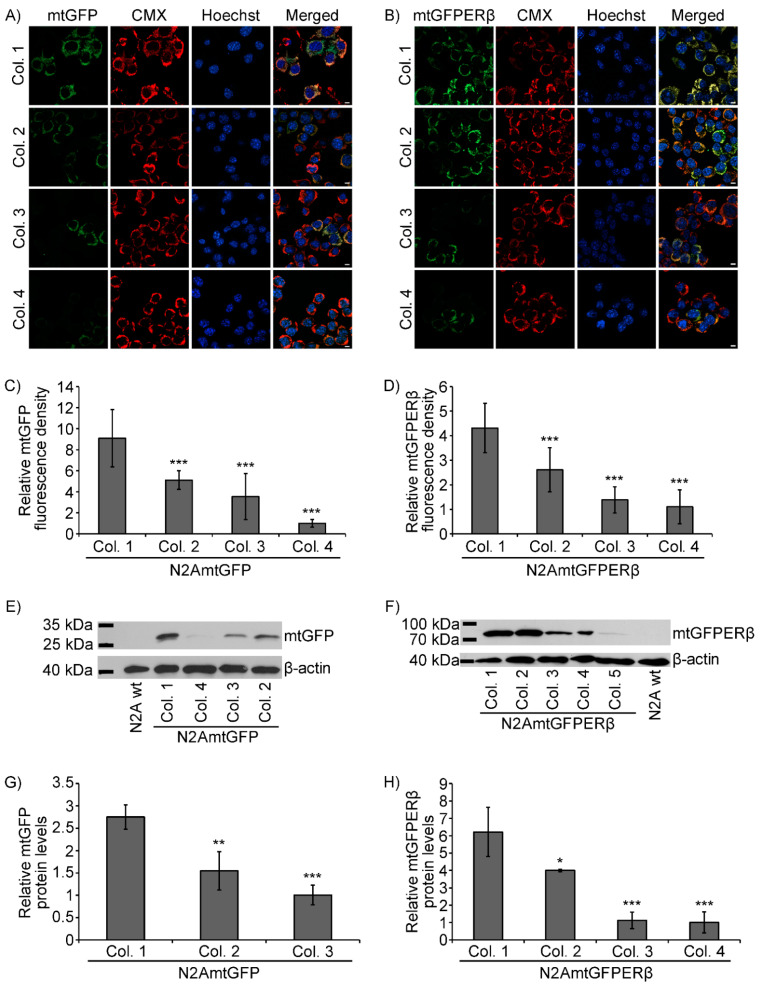
Characterization of the N2AmtGFP, N2AmtGFPERβ stable cell lines. Confocal microscopy analysis for the assessment of the GFP expression levels in colonies of the N2AmtGFP (**A**) and N2AmtGFPERβ, (**B**) cells and its colocalization with the CMX mitochondrial dye. Hoechst staining was applied for nuclear staining. Bars indicate 10 μm. Quantification of the relative expression levels of the mitochondrial green fluorescence protein in colonies of the N2AmtGFP and N2AmtGFPERβ cells is presented in (**C**,**D**), respectively. The lowest expression level was set as 1. Data are presented as the mean ± SD; *** *p* < 0.001 (*n* = 30–50), compared to the respective colony 1 of each cell line. Representative images of Western blot analysis of GFP and β-actin (**E**,**F**), and quantification of the GFP expression levels in colonies of the N2AmtGFP and N2AmtGFPERβ cells (**G**,**H**) are presented. Western blot analysis of β-actin was applied for the normalization of the results (**G**,**H**). The lowest GFP expression level, in the respective colony of each cell line was set as 1. Data are expressed as mean ± S.D. (*n* = 3), * *p* < 0.05, ** *p* < 0.01, *** *p* < 0.001, compared to the respective colony 1 of each cell line.

Confocal microscopy single section images of colonies of N2A cells stably overexpressing a mitochondrial targeted GFP (mtGFP) or a mitochondrial targeted GFPERβ protein (mtGFPERβ) are shown in Figure 1A,B, respectively. As is shown in Figure 1, staining of GFP in N2AmtGFP and N2AmtGFPERβ cells (Figure 1A,B) exhibited the same pattern and colocalized with the mitochondrial marker MitoTracker Red CMXRos (CMX), verifying the mitochondrial localization of the expressed mtGFPERβ and mtGFP proteins in the N2AmtGFP and N2AmtGFPERβ cells, respectively. Quantification of the relative expression levels of the fluorescent mtGFP and mtGFPERβ proteins in the respective cell colonies was performed as described in the experimental section and the results are presented in Figure 1C,D. Quantitative colocalization analysis of the GFP fusion proteins and the CMX mitochondrial staining in N2AmtGFP and N2AmtGFPERβ cells revealed a Pearson’s correlation coefficient of 0.75 and 0.62, respectively, verifying the colocalization of the GFP and the mitochondrial CMX staining. Similarly, quantitative colocalization analysis of the GFP fusion proteins and the Hoechst 33342 nuclear staining in N2AmtGFP and N2AmtGFPERβ cells revealed a Pearson’s correlation coefficient of −0.25 and −0.23, respectively, indicating no colocalization of the GFP and the nuclear Hoechst staining (see also Supplementary Figure S1). The pattern of GFPERβ staining did not indicate plasma membrane localization of the receptor. Verification and assessment of the relative expression levels of the mtGFP and mtGFPERβ proteins in colonies of the respective stable cell lines of N2A cells was also achieved by Western blot analysis, using specific anti-GFP antibodies (Figure 1E,F). Quantification of the results is presented in Figure 1 G,H. Thus, as is shown in Figure 1D,H, colony 1 (Col. 1) of the N2AmtGFPERβ cells exhibited the highest mtGFPERβ expression levels and showed approximately two- to five-fold higher mtGFPERβ expression compared to the other positive selected colonies (Figure 1D,H). As regards N2AmtGFP cells, colony 1 of the N2AmtGFP cells showed two- to eight-fold higher expression levels of mtGFP, compared to the respective positive selected colonies (Figure 1C,G).

**Figure 2:**
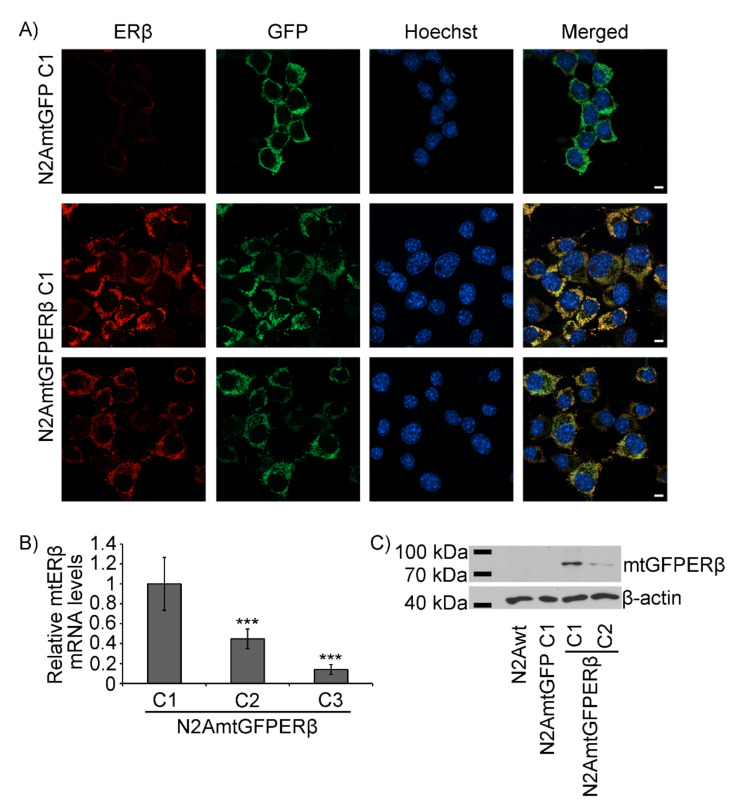
Verification of the mitochondrial ERβ expression in N2AmtGFPEβ cells. (**A**) Confocal colocalization studies in colony 1 of the N2AmtGFP and N2AmtGFPERβ cells. Anti-ERβ antibody and anti-mouse Alexa 568 secondary antibodies were applied for the ERβ detection. Mitochondrial GFP fusion proteins are depicted in green. Nuclear Hoechst staining is depicted in blue. Bars indicate 10 μm. (**B**) Results from real-time PCR analysis for the assessment of the relative human mRNA ERβ levels in colonies of N2AmtGFPERβ cells. β-actin was used as reference gene. The highest mRNA expression level in the respective colony of the N2AmtGFPERβ was set as 1. Data are presented as the mean ± SD; *** *p* < 0.001 (*n* = 4–5), compared to colony 1. (**C**) Western blot analysis of mtGFPERβ, in N2A and in colonies of N2AmtGFP and N2AmtGFPERβ cells. Anti-ERβ and anti-β-actin antibodies were used for ERβ and β-actin detection.

To further confirm the expression and the mitochondrial localization of the GFPERβ protein, immunofluorescence, and Western blot analysis using antibodies against ERβ were performed. Thus, as is shown in Figure 2A, immunofluorescence analysis using antibodies against human ERβ (hERβ) verified the colocalization of the stably expressed hERβ with the mitochondrial GFP staining. Quantitative colocalization analysis of the CMX and ERβ staining revealed a Pearson’s correlation coefficient of 0.84 (see also supplementary Figure S1). Moreover, via Western blot analysis using antibodies against human ERβ, the detection of the GFPERβ fusion protein in colonies of the N2AmtGFPERβ cells, but not in N2A and N2AmtGFP cells, was verified (Figure 2C). Endogenous levels of ERβ in N2A cells (Figure 2A,C) are limited and thus undetectable in accordance with previously reported observations [25,26,27]. The overexpression of mtERβ in N2A mtGFPERβ stable cell lines was also confirmed by assessment of human ERβ mRNA levels. For that purpose, specific primers for the human ERβ gene (ERβ forward: CGGAAGCTGGCTCACTTGCT; ERβ reverse: ATGCCTGACGTGGGACAGGA) (Figure 2B) and primers for β-actin (β-actin forward: TGTGACGTTGACATCCGTAA; β-actin reverse: GCTAGGAGCCAGAGCAGTAA) as a housekeeping gene were applied in real-time PCR analysis. An approximately two- to three-fold increase in mRNA levels of the human ERβ levels in colony 1, compared to that of colony 3 and 2, respectively, was observed (Figure 2B). The differential expression of mRNA levels in colonies of N2AmtGFPERβ cells is in accordance with the differential protein expression levels, presented in Figure 1D,H.

The results from Figure 1 and Figure 2 show that colonies of number 1 (colony 1) of the N2AmtGFPERβ and N2AmtGFP cells showed the highest expression of the mtGFPERβ and mtGFP proteins, exhibiting four- to eight-fold higher expression of the mtGFPERβ and mtGFP proteins, respectively, compared to the respective ones in the others selected colonies. Thus, colonies of number 1 were selected for further analysis as described below.

### 2.2. Effect of mtERβ on Staurosporine-Induced Apoptosis

**Figure 3:**
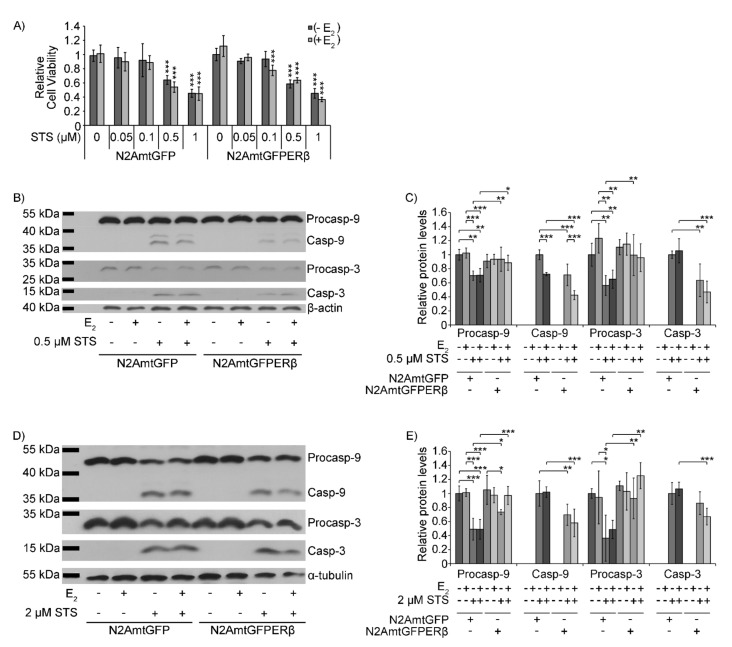
Protective effect of mtERβ on straurosporine-induced apoptosis. (**A**) Assessment of the relative cell viability of N2AmtGFP and N2AmtGFPERβ cells upon treatment with 0.05 to 1 μM staurosporine for 12 h, in the presence or absence of 10^−9^M E2, via MTT analysis. Data were analyzed by two-way ANOVA and expressed as mean ± S.D. (*n* = 4–6), *** *p* < 0.001, compared to the respective vehicle-treated cells of each cell line. Western blot analysis of procaspase-9 and -3 and cleaved caspase-9 and -3, in N2AmtGFP and N2AmtGFPERβ cells, treated with 0.5 μM (**B**) and 2 μM (**D**) staurosporine, in the presence or absence of 10^−9^M E2, for 12 h. Western blot analysis of β-actin was applied for the normalization of the results. Quantification of the results is presented in (**C**) and (**E**) respectively. Data were analyzed by two-way ANOVA and expressed as mean ± S.D. (*n* = 3–4), * *p* < 0.05, ** *p* < 0.01, *** *p* < 0.001.

To assess the possible anti-apoptotic activities of mtERβ, comparative studies applying MTT cell viability assay, as well as Western blot analysis, of caspase-9 and -3 were performed in N2AmtGFPERβ and N2AmtGFP cells, pre-cultured in a hormone-free medium and subsequently treated, or not, with various concentrations of staurosporine, in the presence or absence of 10^−9^ M E2. As is shown in Figure 3A, no statistically significant differences in cell viability were observed between N2AmtGFPERβ and N2AmtGFP cells treated with staurosporine at a range concentration of 0.05 μΜ to 1 μΜ for 12 h. Incubation of the cells with 0.5–1 μM staurosporine in the presence or absence of E2 caused a 30% to 60% reduction in the cell viability of both cell lines, compared to the respective vehicle-treated cells. No statistically significant differences were observed between E2-treated and E2-non treated cells. According to the literature, treatment of N2A cells with 1 μM for 24 h triggers the induction of apoptosis [28]. Thus, further assessment of caspase activation to investigate the effect of the mitochondrial ERβ in mitochondrial-dependent apoptosis was performed. Interestingly, a statistically significant increased reduction in procaspase-9 and procaspase-3 protein levels in N2AmtGFP cells, compared to the respective ones in N2AmtGFPERβ cells, was observed upon treatment of the cells with 0.5 μΜ or 2 μΜ staurosporine (Figure 3B,C and Figure 3D,E, respectively) for 12 h. Accordingly, a statistically significant increased production of activated cleaved caspase-9 in 0.5 μΜ or 2 μM staurosporine-treated N2AmtGFP cells, compared to that in N2AmtGFPERβ cells, was observed (Figure 3B,C and Figure 3D,E, respectively). Similarly, assessment of cleaved caspase-3 protein levels revealed an increased resistance in cleaved caspase-3 production in staurosporine-treated N2AmtGFPERβ cells compared to the respective ones in N2AmtGFP cells. Thus, a statistically significant reduction in cleaved caspase-3 production was observed in N2AmtGFPERβ, compared to that in N2AmtGFP cells, upon treatment with the relative low concentrations (0.5 μΜ) of staurosporine (Figure 3B,C) and upon co-treatment with 0.5 or 2 μΜ staurosporine/10^−9^ M E2 (Figure 3B,C and Figure 3D,E, respectively). As it was expected, the presence of E2 led to suppression of the staurosporine-induced apoptosis in both cell lines only when the cells were treated with relatively low concentrations of staurosporine. Thus, reduced cleaved caspase-9 protein levels in E2/0.5 μΜ staurosporine treated cells compared to those in E2-non treated/0.5 μΜ straurosporine-treated cells (Figure 3B,C) was observed.

### 2.3. Effect of mtERβ on H_2_O_2_-Induced Apoptosis

**Figure 4:**
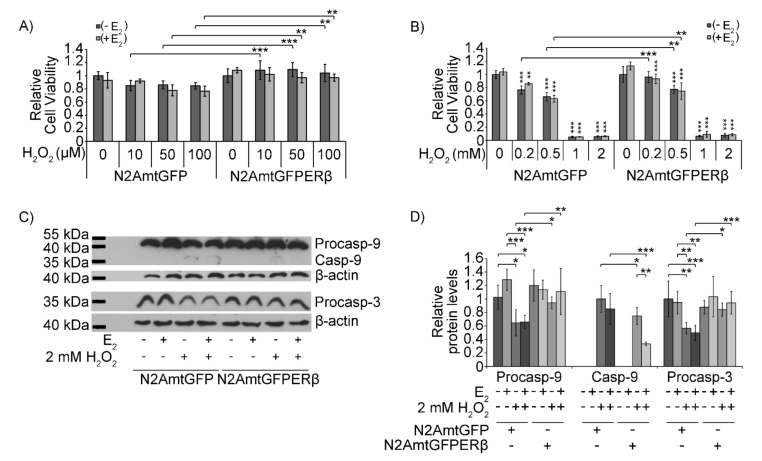
Protective effect of mtERβ on H_2_O_2_-induced apoptosis. Assessment of the relative cell viability of N2AmtGFP and N2AmtGFPERβ cells upon treatment with 10 to 100 μM H_2_O_2_ (**A**) and 0.2 mM to 2 mM H_2_O_2_ (**B**) for 12 h, in the presence or absence of 10^−9^M E2, via MTT analysis. Data were analyzed by two-way ANOVA and expressed as mean ± S.D. (*n* = 4–6), ** *p* < 0.01, *** *p* < 0.001. Vertical asterisks represent statistically significant differences compared to the respective vehicle-treated cells of each cells line, while horizontal lines and the respective asterisks corresponds to statistically significant differences between the two types of cells subjected to the same treatment, as indicated. (**C**) Western blot analysis of procaspase-9 and -3 and cleaved caspase-9 in N2AmtGFP and N2AmtGFPERβ cells, treated with 2 mM H_2_O_2_, in the presence or absence of 10^−9^M E2, for 2.5 h. Western blot analysis of β-actin was applied for the normalization of the results. Quantification of the results is presented in (**D**). Band density of the highest expression level of each analyzed molecule, in the respective set of experiment, was set at 1. Data were analyzed by two-way ANOVA and expressed as mean ± S.D. (*n* = 3–5), * *p* < 0.05, ** *p* < 0.01, *** *p* < 0.001.

In the same context, the role of mtERβ in H_2_O_2_-induced apoptosis was assessed in N2AmtGFP and N2AmtGFPERβ cells. Comparative studies on cell viability and caspase activation were performed for that purpose (Figure 4). MTT cell viability assay revealed a higher resistance (by approximately 20–30%) to the H_2_O_2_-induced reduction in cell viability in N2AmtGFPERβ cells treated with 10 to 500 μΜ H_2_O_2_ for 12 h, compared to the H_2_O_2_-treated N2AmtGFP cells (Figure 3A,B). No statistically significant differences between the N2AmtGFPERβ and N2AmtGFP cells were observed upon treatment with H_2_O_2_ at concentrations higher than 0.5 mΜ. Treatment with 1 and 2 mM H_2_O_2_ for 12 h caused a severe reduction in cell viability that reached 90% compared to control treated cells (Figure 4B). The presence of E2 did not significantly alter H_2_O_2_ effects on cell viability (Figure 4A,B). Western blot analysis of caspase activation upon treatment with 2mM H_2_O_2_ for 2.5 h was also performed. Treatment conditions were chosen based on results from pilot experiments (Supplementary Figure S2). Thus, Western blot analysis of caspase-9 and -3 in 2 mM H_2_O_2_ treated cells for 2.5 h showed a statistically significant increase in cleaved caspase-9 protein levels and a statistically significant increase in the reduction of the pro-caspase-9 and -3 protein levels in N2AmtGFP cells, compared to that in N2AmtGFPERβ cells. These results further substantiate the mtERβ-associated resistance of N2A cells to H_2_O_2_-induced apoptosis (Figure 4C,D). The protective actions of mtERβ are strengthened in the presence of E2, as indicated by the statistically significant reduced production of cleaved caspase-9 in the presence of E2 in H_2_O_2_-treated N2AmtGFPERβ cells compared to that in the N2AmtGFP cells (Figure 4C,D).

### 2.4. Effect of mtERβ on H_2_O_2_-Induced Mitochondrial Impairment in N2A Cells

**Figure 5:**
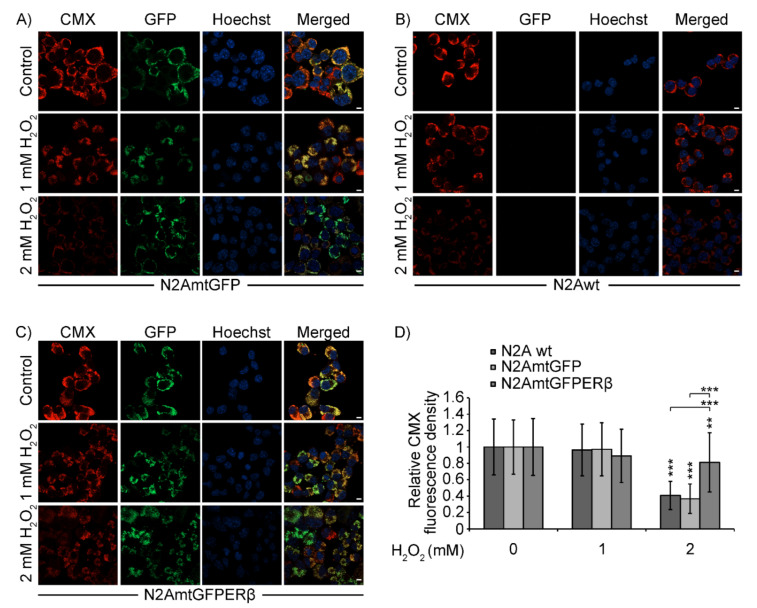
Effect of mtERβ on H_2_O_2_-induced mitochondrial impairment in N2AmtGFP (**A**), N2Awt (**B**), and N2AmtGFPERβ (**C**) cells. (**A**–**C**) Representative confocal microscopy single section images of GFP, CMX (mitochondrial staining) and Hoechst 33342 (nuclear staining). Bars indicate 10 μm. (**D**) Quantification of the relative CMX fluorescence density. Data were analyzed by two-way ANOVA. Relative CMX fluorescence density was expressed as mean ± S.D. (*n* = 30–50), ** *p* < 0.01, *** *p* < 0.001. CMX fluorescence density at vehicle treated N2A cells was set at 1. Vertical asterisks represent statistically significant differences compared to the respective vehicle treated cells of each cells line, while horizontal lines and the respective asterisks correspond to statistically significant differences between cells from different cell lines subjected to H_2_O_2_ treatment, as indicated.

The effect of mtERβ on H_2_O_2_-induced mitochondrial impairment was also assessed by comparative measurements of Mitotracker red CMX staining in H_2_O_2_-treated N2A, N2AmtGFP, and N2AmtGFPERβ cells. CMX is a red fluorescence indicator used to detect oxidation–reduction reactions, which principally occur in active mitochondria of live cells. Thus, CMX stains mitochondria in live cells and its accumulation is dependent upon mitochondrial membrane potential [29,30,31]. As is shown in Figure 5, treatment of N2A, N2AmtGFP, and N2AmtGFPERβ cells with 2 mM H_2_O_2_ for 2.5 h revealed a statistically significant reduction in mitochondrial staining of H_2_O_2-_treated N2A and N2AmtGFP cells compared to that in N2AmtGFPERβ cells.

### 2.5. Effect of Mitochondrial ERβ on ATP Content

**Figure 6:**
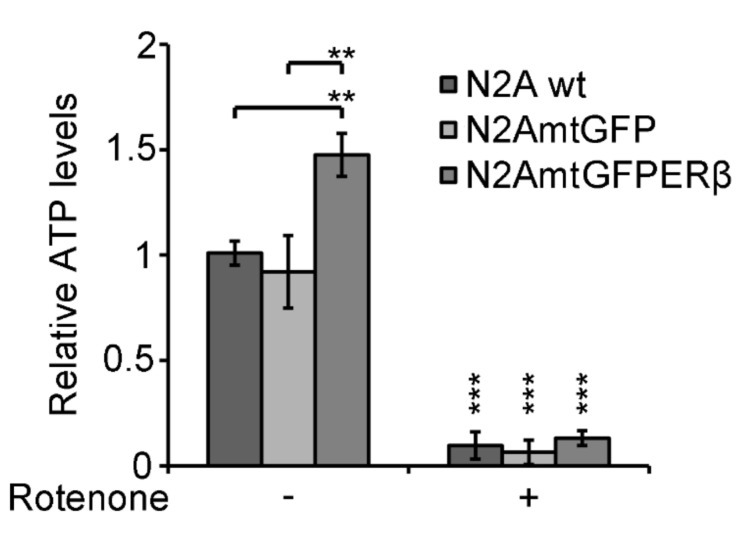
Effect of the mitochondrial ERβ on ATP levels in N2A, N2AmtGFP, and N2AmtGFPERβ cells. Equal numbers of N2A, N2AmtGFP, and N2AmtGFPERβ cells were plated on six well plate. At 70–80% confluency, cells were incubated with 0.2 μΜ rotenone (diluted in DMSO) for 16 h. Control cells were treated with equal volumes of DMSO. Subsequently, cells were washed in PBS, harvested, and subjected to ATP measurements, applying ENLITEN ATP bioluminescence assay as described in experimental section. Results are expressed as relative luciferase units, normalized against relative protein levels and represent the mean ± SD of three independent experiments. ** *p* < 0.01, *** *p* < 0.001. ATP levels at vehicle-treated N2A cells were set at 1. Vertical asterisks represent statistically significant differences compared to the respective vehicle-treated cells of each cell line, while horizontal lines and the respective asterisks correspond to statistically significant differences between cells from different cell lines.

To assess whether mitochondrial ERβ protective activity is associated with its involvement in the regulation of mitochondrial energy production, assessment of the ATP levels in N2A, N2AmtGFP, and N2AmtGFPERβ cells was performed in the presence or absence of 0.2 μΜ rotenone, which is an inhibitor of complex I of the mitochondrial respiratory chain. Treatment with rotenone was applied to evaluate the mitochondrial origin of ATP. As is shown in Figure 6, N2AmtGFPERβ exhibited approximately a 1.5-fold statistically significant increase in ATP content compared to that in the N2AmtGFP and N2A cells. Preincubation of the cells with 0.2 μM rotenone for 16 h caused elimination of ATP production in both cell lines, validating the mitochondrial origin of ATP.

## 3. Discussion

In this study, the direct involvement of the mitochondrial estrogen receptor beta (mtERβ) in the activation of the mitochondrial defense antioxidant and anti-apoptotic activities is demonstrated. Accumulated evidence substantiates the regulatory and protective actions of estrogens on the brain, affecting both glial and neuronal physiology [17,21,32,33,34,35]. Particularly, the regulation of neuronal mitochondrial functions by estrogen is widely accepted [36,37]. Most of these actions are mediated by the nuclear or the membrane estrogen receptors [6,13]. In addition, considerable amounts of experimental data showed ER-independent estrogen actions [17,35], or suggested the involvement of the mitochondrial estrogen receptors, especially ERβ, in these actions [38,39,40,41,42,43]. Thus, direct actions of estrogens on mitochondria are proposed to be exerted via their mitochondrial-localized estrogen receptors. This hypothesis is based on data showing mitochondrial localization of ΕRβ in a variety of neuronal cells and tissues [10,11,44]. In addition, data showing the interaction of ERβ with the mitochondrial DNA [45], the mitochondrial Hydroxyacyl-CoA Dehydrogenase Trifunctional Multienzyme Complex Subunit Beta (HADHB) fatty acid oxidation enzyme, [46,47], the mitochondrial adenosine triphosphatase (ATPase) [47,48], and the pro-apoptotic BAD protein [49,50] indicate possible involvement of the mitochondrial ERβ in the regulation of mitochondrial transcription, energy metabolism and apoptosis.

Mitochondrial ATP production by oxidative phosphorylation is critical for neurons to meet their energy demands and survive. In addition, mitochondrial impairment, induced by excessive cytoplasmic Ca^2+^ loading or increased ROS generation, could lead to mitochondrial membrane potential depolarization, mitochondrial swelling, release of apoptotic factors, caspase activation, and eventually the induction of apoptosis and cell death. Thus, mitochondrial dysfunction is closely related to neuronal viability and neurodegeneration [38]. A non-genomic effect of E2 on the regulation of Ca2+ transport in brain mitochondria and the involvement of ERβ in this action is proposed [51,52]. Thus, the verification and elucidation of the direct actions of estrogens on mitochondria is of great interest. The presence of ERβ in mitochondria could establish a direct control of mitochondrial activities by estrogens and could ensure estrogens’ actions on the integration and orchestration of nuclear and mitochondrial functions. Due to the widely accepted neuroprotective actions of estrogens and to the great importance of the preservation of mitochondrial functions in neurodegenerative diseases [17,22,53], the delineation of the putative mitochondrial estrogen receptors’ anti-apoptotic and anti-oxidant actions could uncover novel therapeutic targets for those high-risk disorders.

An obstacle to the verification of the direct mitochondrial ERβ actions, and to the delineation of the biochemical mechanisms of its actions, is the questionable availability of specific antibodies against ERβ [54]. To overcome this impediment, neuroblastoma N2A cell lines stably overexpressing a mitochondrial-targeted GFPERβ (N2A mtGFPERβ) were generated, and the cells were further analyzed for their resistance to apoptotic and oxidative stimuli in comparative studies. Overexpression of the mtGFPERβ protein in the selected G418-resistant colonies was confirmed by Western blot analysis, using antibodies against GFP and antibodies against ERβ. Results were verified by real-time PCR using ERβ-specific primers. Moreover, the mitochondrial localization of the expressed mtGFPERβ protein was validated in colocalization studies of the GFP and ERβ proteins with the mitochondrial CMX dye, applying immunofluorescence analysis and confocal microscopy studies. Verification of the expression and the mitochondrial localization of the green fluorescence protein in N2AmtGFP control cells was also achieved. Colonies exhibiting the highest expression of the mtGFPERβ and mtGFP proteins were used in this study.

Mitochondrial ROS production and induction of apoptosis is closely related to many degenerative diseases, including neurodegeneration [55,56,57]. In the current study, we showed mitochondrial ERβ-dependent resistance in the induction of apoptosis upon treatment of the cells with either the apoptotic stimulus staurosporine or the oxidative stress stimulus H_2_O_2_. Thus, assessment of caspase-9 and caspase-3 activation, applying Western blot analysis, revealed decreased production of cleaved caspase-9 and -3, and reduced proteolytic degradation of procaspase-9 and -3 in N2AmtGFPERβ cells compared to that in N2AmtGFP cells when treated either with 0.5 to 2 μM staurosporine or 2 mM H_2_O_2_. Moreover, upon treatment with relatively low concentrations of apoptotic stimuli, the presence of E2 strengthened the mtERβ-dependent resistance in the induction of apoptosis in N2AmtERβ cells, whereas the inability of E2 to reverse this effect in control N2AmtGFP cells was observed. This observation indicates that the presence of mtERβ is adequate to offer protection against apoptotic stimuli, but its activity is also at least in part estrogen-dependent. The protective antioxidant activities of the mtERβ were also verified in fluorescence microscopy studies of the CMX mitochondrial dye, which is incorporated exclusively in functionally active mitochondria [29,30]. Upon H_2_O_2_-induced oxidative stress, the percentage of sustained functionally active mitochondria was higher in mtERβ-overexpressing N2A cells, compared to control N2A and N2AmtGFP cells. This observation is in agreement with previous studies showing anti-apoptotic and antioxidant activities of estrogens in mitochondria, which is proposed to be mediated by nuclear and membranous ERs, as well as by estrogen receptor-independent estrogen action [58,59,60,61,62]. The results from this study provide evidence for the direct involvement of the mitochondrial ERβ in this process. Thus, since endogenous ERβ levels in N2A cells are limited [25,27], the results from this study indicate that estrogens’ protective actions are also mediated, at least in part, via the mitochondrial estrogen receptor beta.

In accordance with results from Western blot analysis and fluorescence microscopy studies, MTT cell viability assay confirmed the protective effect of the mtERβ overexpression in cells treated with H_2_O_2_ at a concentration range of 10–500 μM, even upon conditions of a longer incubation time (12 h/2.5 h). This effect was not detectable at higher concentrations of H_2_O_2_, possibly due to severe induction of the oxidative stress, which could not be reversible by the protective activity of mtERβ. No statistically significant differences between N2AmtGFPERβ and N2AmtGFP cell viability were also observed in cells treated with staurosporine. Nevertheless, results from the Western blot analysis uncovered the protective actions of mtERβ, as was shown by the reduced activation of caspase-9, which corresponds to the initial steps of mitochondrial-induced apoptosis.

Mitochondria are the main energy suppliers of the cell. The maintenance of functional, active mitochondria ensures adequate energy production, which is essential for neuron survival and the prevention of degenerative processes that lead to neurodegenerative diseases and aging [24]. Thus, factors that ensure and reinforce energy production are crucial regulators of neuronal viability and function. In this context, mtERβ can be considered such a valuable factor, as indicated by our observations showing increased ATP content in N2AmtGFPERβ compared to that in N2AmtGFP cells. The elimination of ATP level by rotenone substantiates its mitochondrial origin, further supporting the involvement of mitochondrial ERβ in this action.

Thus, anti-apoptotic and antioxidant activities of the mitochondrial-localized ERβ could be attributed to its proposed actions on the mitochondrial transcriptional activation, and thus oxidative phosphorylation enzyme activation [12,42], that ends up in an increase in ATP production and a reduction in ROS production. In addition, anti-apoptotic and antioxidant activities of mtERβ can also be mediated via their involvement in the regulation of the activity of mitochondrial apoptosis-associated and antioxidant molecules, such as BAD and MnSOD [4,50,63,64], via mtERβ interaction with other mitochondrial components or via its interfering with other estrogen-dependent or estrogen-independent signaling pathways, such as Ca2+ modulating effects [11,51,52]. Moreover, since E2 is known to activate the synthesis of many nuclear-encoded antioxidant enzymes, namely MnSOD, thioredoxin, and glutathione peroxidase [65,66,67,68], the involvement of mtERβ in the orchestration of the synthesis of nuclear-encoded mitochondrial antioxidant- and apoptosis-associated molecules is also an interesting issue that remains to be explored.

To sum up, the results from this study revealed an increased resistance to staurosporine- and H_2_O_2_-induced apoptotic stimuli in N2A cells overexpressing a mitochondrial-targeted estrogen receptor beta, as indicated by the reduced activation of caspase-9 and -3, the increased cell viability and ATP production, and the increased resistance to mitochondrial impairment in the presence or absence of E2. Thus, the direct involvement of mtERβ in the antioxidant and anti-apoptotic E2 actions is documented, rendering ERβ a promising therapeutic target for mitochondrial-related degenerative diseases.

## 4. Materials and Methods

### 4.1. Chemicals

Dulbecco’s modified eagle medium (DMEM), trypsin, fetal bovine serum (FBS), Lipofectamin 2000, and MitoTracker Red CMXRos (CMX) were obtained from Thermo Fisher Scientific (Thermo Fisher Scientific GmbH, Basel, Switzerland). Molecular weight markers, complete protease cocktail inhibitors, and Western blotting luminol reagent were purchased from Thermo Fisher Scientific, Roche (Mannheim, Germany) and Santa Cruz Biotechnology (Santa Cruz Biotechnology, Inc., Dallas, TX, USA), respectively. Geneticin (G418) was provided by Calbiochem (Merck, Kenilworth, NJ, USA). All other chemicals, including Hoechst 33342, staurosporine, estradiol (E2), and H_2_O_2_, were purchased from Sigma-Aldrich (St. Louis, MO, USA).

### 4.2. Antibodies

Monoclonal antibodies against human ERβ (B-3) and α-tubulin were provided by Santa Cruz Biotechnology. Monoclonal antibodies against β-actin were provided by Sigma Aldrich. Monoclonal antibodies against caspase-9 and -3 and polyclonal antibodies against cleaved caspase-3 were provided by Cell Signaling (Cell Signaling Technology, Danvers, MA, USA). Monoclonal antibodies against GFP were provided by Roche. Horseradish peroxidase (HRP)- and Alexa 568-conjugated secondary antibodies were purchased from Thermo Fisher Scientific.

### 4.3. Cell Culture—Generation of N2A Cells Stably Expressing a Mitochondrial Targeted GFPERβ Fusion Protein

Mouse N2A neuroblastoma cells (obtained from the American type culture collection—ATCC) were maintained in low glucose (1 g/L) DMEM, supplemented with 10% FBS, 2 mM glutamine, and 50 units/mL penicillin/streptomycin (DMEM complete medium). Cells were grown at 37 °C in a humidified atmosphere with 5% CO_2_. For stable cell lines generation, at day 1 before transfection, N2A cells were plated in 60 mm culture dishes so that they reached 60–70% confluency at the time of transfection. To obtain stable expression of the human mitochondrial ERβ, the human estrogen receptor beta gene (AB006590) [69] was inserted into the pEGPC2 vector (Clontech-Takara, Mountain View, CA, USA), in frame with the enhanced green fluorescence protein (EGFP) gene between Xho1 and BamH1 sites, to produce the pEGFPC2ERβ vector. Subsequently, the following sequence: atggctcagcgacttcttctgaggaggttcctggcctctgtcatctccaggaagccctctcagggtcagtggccacccctcacttccagagccctgcagaccccacaatgcagtcctggtggcctgctgtaacacccaacccagcccggacaatatacaccacgaggatctccttgaca, which encodes a mitochondrial targeting peptide, was inserted in frame with the EGFP Gene between the NheI and AgeI sites of the pEGFPC2ERβ construct to produce the pmtEGFPERβ construct. The produced construct was transfected into the N2A cells using Lipofectamin 2000 (Thermo Fisher Scientific GmbH, Europe), according to the manufacturer’s instructions. Control cells (N2AmtGFP cells) were prepared by transfection of the pmtEGFPC2 construct [70]. After 36 h incubation at 37 °C, cells were trypsinized and passed at a 1:14 dilution in selective growth medium [DMEM supplemented with 10% (*v/v*) FBS, containing 1.5 mg/mL G418]. On the next day, the medium in all plates was replaced with fresh selective medium and cells were cultured for 2 weeks. G418-resistant colonies were expanded, cloned independently, and analyzed by immunofluorescence and Western blot analysis to confirm the expression of the mitochondrial-GFP (mtGFP) and -GFPERβ (mtGFPERβ) proteins in the N2AmtGFP and N2AmtGFPERβ cells, respectively.

### 4.4. Western Blot Analysis

Western blot analysis of molecules involved in mitochondria-mediated apoptosis, such as caspase-9 and caspase-3, was performed in extracts from N2AmtGFP and N2AmtGFPERβ cells, treated, or not, with 0.5–2 μΜ staurosporine or 2 mM H_2_O_2_, in the presence or absence of estradiol, using specific antibodies. Briefly, cells plated on 6 well plates were cultured for 40–48 h in low glucose DMEM without phenol red, supplemented with 10% dextran-coated charcoal stripped fetal bovine serum. Then, cells were incubated with 0.5–2 μΜ staurosporine or 2 mM H_2_O_2_ at various time points, as indicated in the results section. After the washing of the cells with phosphate buffer saline (PBS), cells were lysed in lysis buffer (20 mM Tris pH: 7.5, 250 mM NaCl, 0.5% Triton, 3 mM EDTA) supplemented with cocktail protease inhibitors. Protein determination, electrophoresis and Western blot analysis were performed as previously described [71]. Quantification of the results was carried out by applying ImageJ (1.52 p) analysis (NIH, Bethesda, MD, USA).

### 4.5. Immunofluorescence—Confocal Microscopy

Cells grown on coverslips for 48 h in DMEM complete medium were further incubated for 30 min at 37 °C with 200 nM CMX. In the case of H_2_O_2_ treatment, cells were treated with 1 mM or 2 mM H_2_O_2_ for 2.5 h at 37 °C in the presence of 200 nM CMX. Subsequently, cells were washed 3 × 5 min with PBS, fixed for 10 min in ice-cold methanol, and transferred to ice-cold acetone for 2 min. For nuclear staining, specimens were incubated with 1 μg/mL Hoechst 33,342 in PBS for 30 min at room temperature. Antibodies against ERβ (B-3, Santa Cruz Biotechnology, Dallas, TX, USA) were applied for ERβ detection. Secondary anti-mouse Alexa 568-conjugated antibodies were used. The specimens were washed in PBS and mounted in anti-fading medium. Images were taken using an inverted laser-scanning confocal microscope (Zeiss laser scanning microscope 800, Zeiss LSM 800, Zeiss, Jena, Germany). Image analysis and quantification of fluorescence staining were performed as previously described [25] using the ImageJ (1.52 p) analysis program. Briefly, the total corrected fluorescence of area of interest (TCF) = integrated density − (selected area × mean fluorescence of background readings) was calculated. The TCF of treated cells was then normalized against the mean TCF of the relative control cells (Relative Fluorescence Density). Quantitative colocalization analysis of the GFP fusion proteins and the CMX and Hoechst staining, and of the ERβ and the CMX staining, was performed by measuring the Pearson’s correlation coefficient, applying the ZEN Zeiss LSM 800 software, according to manufacturer’s instructions.

### 4.6. Cell Viability Assay

In order to investigate the role of mtERβ on the cell viability under stress conditions, the MTT assay [72] was performed. Cells of the two stable cell lines were plated on 96-well plates and were cultured in low glucose DMEM without phenol red, supplemented with 10% dextran-coated charcoal stripped fetal bovine serum. After 40–48 h, the cells were incubated with various concentrations of staurosporine or H_2_O_2_ in the presence or absence of E2 for 12 h, as indicated in the results section. Then, the cells were incubated with 0.5 mg/mL MTT for 3 h at 37 °C and 5% CO_2_ and the formed formazan crystals were dissolved with isopropanol. The color intensity at 570 nm (reference filter at 690 nm) was measured using a multimode plate reader (EnSpire, PerkinElmer, UK). Relative cell viability corresponds to absorbance values of treated cells normalized against absorbance values of the relative control cells.

### 4.7. ATP Measurements

ATP contents in N2A, N2AmtGFP, and N2AmtGFPERβ was measured as previously described [70]. Briefly, cells grown in DMEM complete medium were thrypsinized and plated in a 6-well plate in hormone-free DMEM medium for 48 h. Subsequently, cells were treated in the presence or absence of 0.2 μΜ rotenone for 16 h. Cells were washed in PBS, harvested and centrifuged at 800× *g*. ATP from the cell pellet was extracted with 1% tricloroacetic acid and ATP content was measured in a Luminometre (LB 9508, Berthold, Europe) by bioluminescence using the luciferin–luciferase reaction kit (Enliten, Promega Coorporation, Madison, WI, USA) following the manufacturer’s instructions.

### 4.8. Statistical Analysis

All results are expressed as mean ± SD. Data were analyzed by two-way ANOVA followed by Tukeys’s post-hoc test using StatPlus LE 7.3.0 software. Differences were considered significant at a two-tailed *p* value < 0.05.

## Supplementary Materials

The following are available online at https://www.mdpi.com/article/10.3390/ijms22147620/s1.
