# Supplementary material for: Anti-Apoptotic and Antioxidant Activities of the Mitochondrial Estrogen Receptor Beta in N2A Neuroblastoma Cells
Source: Int J Mol Sci. 2021 Jul 16;22(14):7620. doi: 10.3390/ijms22147620 (PMC8306648; doi:10.3390/ijms22147620)
Supplement: Supplementary file 1 [file ijms-22-07620-s001.zip › ijms-1271168-supplementary.pdf]

Supplementary Data

Anti-apoptotic and Antioxidant Activities of the Mitochondrial Estrogen Receptor Beta in N2A Neuroblastoma Cells

Ioannis Tsialtas, Achilleas Georgantopoulos, Maria E. Karipidou, Foteini D. Kalousi, Aikaterini G. Karra, Demetrios D. Leonidas and Anna-Maria G. Psarra *

Department of Biochemistry and Biotechnology, University of Thessaly, 41500 Larissa, Greece; tsialtasj@gmail.com (I.T.), ageorgant@bio.uth.gr (A.G.); mkaripidou@uth.gr (M.E.K.); fokalous@bio.uth.gr (F.D.K.); aikaterini.g.karra@gmail.com (A.G.K.); ddleonidas@bio.uth.gr (D.D.L.)

* Correspondence: ampsarra@bio.uth.gr; Tel.: +30-2410-565221

Supplementary Figure 1 (Figure S1)

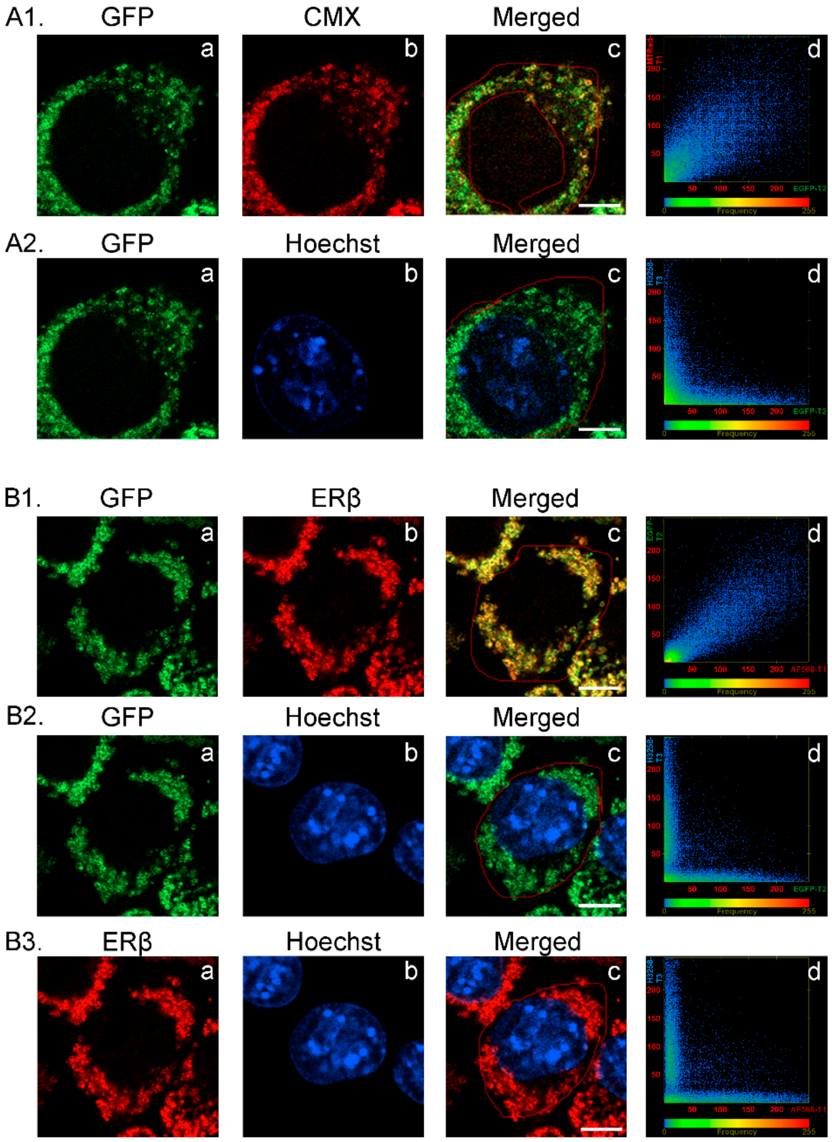

Pixel Count	28106
Area (μm²)	275666
Pearson	0.62
Manders	0.86

Pixel Count	212375
Area (μm²)	36906
Pearson	-0.23
Manders	0.37

Pixel Count	20480
Area (μm²)	200870
Pearson	0.84
Manders	0.93

Pixel Count	4879
Area (μm²)	47854
Pearson	-0.30
Manders	0.46

Pixel Count	6698
Area (μm²)	65695
Pearson	-0.38
Manders	0.39

Figure S1. Quantitative colocalization analysis of the mtERβ and the CMX and/or the Hoechst staining. Quantitative colocalization analysis of A1) GFP and CMX staining, A2) GFP and Hoechst 33342 staining, in CMX and Hoechst stained N2AmtGFPERβ cells of Fig. 1 and of B1) ERβ and CMX staining, B2) GFP and Hoechst staining, and B3) ERβ and Hoechst staining in ERβ, CMX, and Hoechst stained N2AmtGFPERβ cells of Figure 2. Representative singe section images of GFP, ERβ CMX and Hoechst staining are presented. Region of interest (ROI) is indicated in red. Bars indicate 10 µm. Screenshots of the "Colocalization" view showing the graphical scatter plot (d) and the numerical results of the colocalization analysis are presented. Quantitative colocalization analysis was performed, by measuring the Pearson's correlation coefficient and the Mander's overlap coefficient, applying the ZEN Zeiss LSM 800 software, according to manufacturer's instructions.

Supplementary Figure 2 (Figure S2)

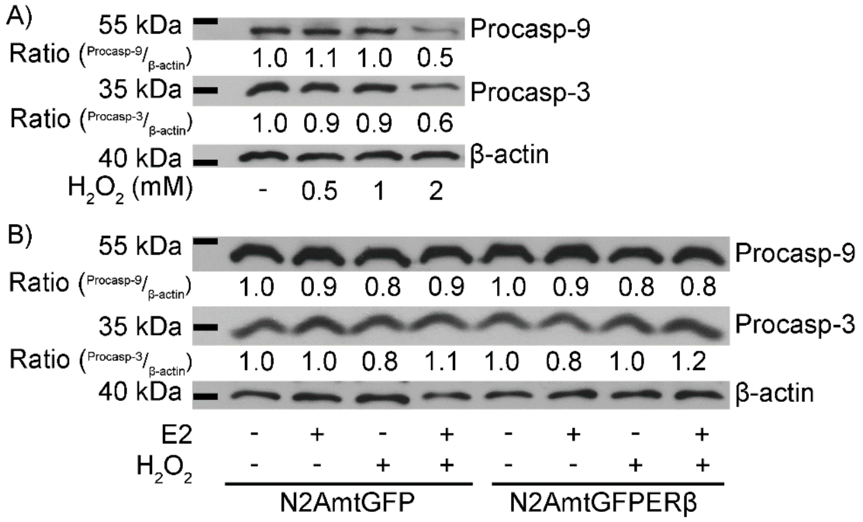

Figure S2. Dose and time dependent effect of H_2O_2 treatment on induction of apoptosis in N2A cells. Pilot studies for setting up the experimental conditions for the characterization of the H_2O_2-induced apoptosis in N2A cells. A) Dose dependent effect of H_2O_2 on caspase -9 and caspase -3 activation in N2A cells. Treatment of N2A cells with 0.5, 1 and 2 mM H_2O_2 for 2.5 h was applied. Caspase -9 and -3 activation was observed upon treatment of N2A with 2mM for 2.5 hrs, as indicated by the reduction in procaspase -9 and -3 levels. B. No remarkable decrease in procaspase -9 and -3 level was observed upon treatment of N2AmtGFP and N2AmtGFPERβ with 1 mM H_2O_2 for 2.5 h. Relative ratio protein levels of procaspase -9 and -3 normalized against the respective β-actin protein levels are presented.
